# Circulating and Tissue Galectin-3 in Gastrointestinal Inflammation: Clinical Significance and Biomarker Potential

**DOI:** 10.3390/cells14191521

**Published:** 2025-09-29

**Authors:** Vesna Brzački, Andriana Jovanović, Andrija Rančić, Snežana Tešić-Rajković, Gordana Petrović, Ivan Nagorni, Marko Stojanović, Elena Stanković, Stefan Momčilović

**Affiliations:** 1 Gastroenterology and Hepatology Clinic, University Clinical Center Niš, 18000 Niš, Serbia; vesna.brzacki@medfak.ni.ac.rs (V.B.); andrija.m.rancic@gmail.com (A.R.); snezanatesic@yahoo.com (S.T.-R.); gordana.petrovic@medfak.ni.ac.rs (G.P.); ivannagorni@gmail.com (I.N.); marcss994@gmail.com (M.S.); stankovicelena008@gmail.com (E.S.); 2 Department of Internal Medicine, Faculty of Medicine, University of Niš, 18000 Niš, Serbia; 3 Clinic for Nephrology, University Clinical Center Niš, 18000 Niš, Serbia; anchej89@gmail.com; 4 Plastic and Reconstructive Surgery Clinic, University Clinical Center Niš, 18000 Niš, Serbia

**Keywords:** Galectin-3, immunomodulation, signaling pathways, gastrointestinal inflammation, Galectin-3 inhibition

## Abstract

Galectins represent a family of widely expressed lectins that have the ability to bind β-galactoside in modulating “cell-to-cell” and “cell-to-matrix” interactions in all organisms. These proteins are expressed in many inflammatory cells, such as macrophages, and depending on the inflammatory environment, they promote pro-inflammatory or anti-inflammatory responses. Galectin-3 (Gal-3) is predominantly located in the cytoplasm, but, as noted, it has also been detected in the nucleus, on the cell surface and in the extracellular environment, which indicates the multifunctionality of this molecule. It has been shown in many studies that Gal-3 is involved in immune regulation, fibrosis, and tissue remodeling, making it an important player in disorders such as inflammatory bowel disease (IBD), non-alcoholic steatohepatitis (NASH), and liver fibrosis. In IBD, this protein is associated with activation of the NLRP3 inflammasome, contributing to chronic intestinal inflammation. Also, in primary biliary cholangitis and autoimmune hepatitis, Gal-3 potentiate development of fibrosis through fibroblast-to-myofibroblast transition and extracellular matrix deposition, while in liver fibrosis, it is upregulated in hepatic stellate cells and macrophages, promoting fibrosis and inflammation. Studies show that Gal-3 inhibition reduces fibrosis and inflammation, making it a promising therapeutic target.

## 1. Introduction

**Figure 1:**
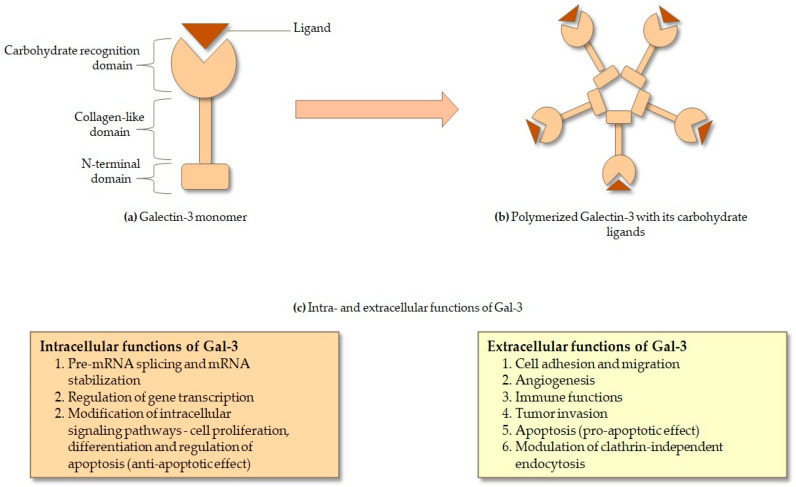
Schematic representation of the structure (**a**), (**b**) and function (**c**) of Gal-3.

Galectin-3 (Gal-3) is a unique member of the galectin family of β-galactoside-binding lectins, distinguished by its chimeric structure comprising a C-terminal carbohydrate recognition domain (CRD) and an N-terminal proline- and glycine-rich domain that mediates protein–protein interactions [1,2]. Unlike the prototype and tandem-repeat galectins, Gal-3 is the only chimera-type galectin, endowing it with exceptional structural flexibility and functional diversity (Figure 1) [1,3]. The CRD consists of approximately 130 amino acids forming a β-sandwich fold with two anti-parallel β-sheets, where the canonical sugar-binding groove specifically recognizes N-acetyllactosamine and related β-galactosides [4,5]. The N-terminal domain contains repeating PGAYPG motifs, which enable self-association into oligomers and the formation of lattice-like structures upon ligand engagement [6,7]. This oligomerization is essential for Gal-3′s ability to cross-link cell-surface glycoconjugates, a property critical for stabilizing receptor clustering and enhancing downstream signaling [8,9]. In line with this, Gal-3 has been implicated in the regulation of desmosomal cadherin steady-state levels, as well as the stabilization of several cell-surface receptors such as the epidermal growth factor receptor (EGFR) and transforming growth factor β receptor (TGF-βR). However, this effect is not universal, since Gal-3 has also been reported to increase the mobility of N-cadherin and to promote the internalization of β1 integrin from the cell surface [8].

Post-translational modifications further diversify Gal-3′s functions. Phosphorylation at serine-6 regulates its intracellular localization, secretion, and anti-apoptotic functions, while cleavage by matrix metalloproteinases (MMPs) generates truncated forms that retain lectin activity but lack oligomerization capacity, thus altering biological effects [10,11]. Gal-3 has both intracellular and extracellular functions (Figure 1): in the cytoplasm it modulates apoptosis and endocytic trafficking, in the nucleus it regulates pre-mRNA splicing, and once secreted it interacts with extracellular matrix (ECM) proteins and cell-surface receptors [12,13,14]. Secretion occurs through a non-classical pathway independent of the endoplasmic reticulum (ER)-Golgi system, underscoring its unconventional biology [15]. Glycan microarray analyses have revealed that Gal-3 preferentially binds poly-N-acetyllactosamine chains on glycoproteins such as integrins, laminins, and growth factor receptors, supporting its role in cell adhesion, migration, and signaling [16,17].

From an evolutionary perspective, Gal-3 is highly conserved among vertebrates, highlighting its fundamental role in host defense, development, and tissue homeostasis [18]. It is expressed in a wide range of tissues, with particularly high levels in immune cells, epithelial barriers, and fibrotic organs, reflecting its context-dependent functions [19]. Its biochemical versatility allows Gal-3 to operate as both a soluble lectin and a structural scaffold, orchestrating multivalent interactions between glycans and proteins. This dual capacity to recognize carbohydrates and to oligomerize distinguishes Gal-3 from other lectins and positions it as a central hub in glycan-mediated communication [20].

**Figure 2:**
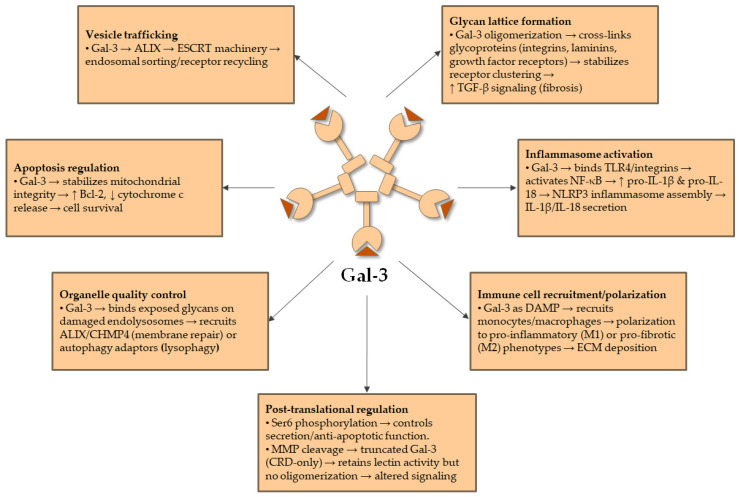
Major functions and signalling pathways of Gal-3. ↑—increased; ↓—decreased.

Functionally, Gal-3 operates across several cellular compartments and biological contexts (Figure 2).

Extracellularly, Gal-3 forms glycan lattices that stabilize and potentiate receptor signalling, such as prolonging TGF-β receptor activation in fibroblasts and stellate cells, thereby enhancing pro-fibrotic transcriptional responses [6,8]. By binding to glycosylated immune receptors including TLR4 and integrins, Gal-3 activates NF-κB-dependent pathways and facilitates NLRP3 inflammasome assembly in macrophages, leading to enhanced production of IL-1β and IL-18 [9]. Acting as a damage-associated molecular pattern (DAMP), Gal-3 recruits and polarizes monocytes/macrophages toward pro-inflammatory and pro-fibrotic phenotypes, amplifying chronic inflammation and ECM deposition [1,12].

Intracellularly, Gal-3 exerts regulatory control over vesicle trafficking, apoptosis, and organelle quality control. By interacting with adaptor proteins such as ALG-2-interacting protein X(ALIX), it orchestrates endosomal sorting and receptor recycling, thereby influencing secretion and surface receptor dynamics. Gal-3 also detects damaged endolysosomal membranes by binding exposed glycans, subsequently recruiting ESCRT machinery (e.g., ALIX, CHMP4) for membrane repair or autophagy adaptors for lysophagy when repair is unsuccessful [14,15]. Additionally, Gal-3 modulates apoptosis by stabilizing mitochondrial integrity and interacting with anti-apoptotic factors, promoting survival of fibroblasts and epithelial cells in stress conditions [10]. Collectively, these extracellular and intracellular functions position Gal-3 as a critical regulator of cellular homeostasis, immune activation, and fibrogenesis [2,3,6].

Given its structural uniqueness and broad mechanistic impact, Gal-3 has emerged as a pivotal mediator linking carbohydrate recognition with signalling networks in health and disease. Elevated Gal-3 expression contributes to pathological processes such as chronic inflammation, fibrosis, and tumor progression, whereas its absence or reduction can impair epithelial repair and immune resolution [2,7,12]. As such, Gal-3 represents both a mechanistic biomarker and a therapeutic target, bridging biochemical properties with clinically relevant signalling pathways.

To date, increased Gal-3 expression has been documented in many pathological conditions, such as gastrointestinal inflammatory diseases, heart and kidney diseases, diabetes mellitus, viral infections, autoimmune diseases, neurodegenerative disorders, and tumor formation [21,22,23].

## 2. The Role of Galectin-3 in Inflammation and Fibrosis

The role of Gal-3 in the inflammatory process has been extensively discussed in many studies. However, in the light of current knowledge, Gal-3 plays a complex and sometimes contradictory role in inflammation. Depending on the cellular context and type, Gal-3 can act as a pro-inflammatory mediator by promoting immune cell recruitment and stimulating pro-inflammatory cytokine production, or as an anti-inflammatory factor by suppressing immune cell activation and inducing apoptosis [24]. Besides its presence in epithelial and endothelial cells, this lectin is also found in various immune cells participating in the inflammatory response, including macrophages, monocytes, dendritic cells, eosinophils, mast cells, natural killer cells, T lymphocytes, and activated B lymphocytes, allowing it to modulate their functions through multiple signaling pathways [24,25].

Despite the fact that it can aid in resolving inflammation by facilitating apoptotic neutrophil clearance, Gal-3 predominantly exerts pro-inflammatory effects [26]. In acute responses, this lectin promotes monocyte/macrophage recruitment, neutrophil clearance, opsonization of apoptotic neutrophils, and mast cell degranulation. In macrophages and epithelial cells, Gal-3 binds to galactoside-containing membrane remnants from vacuoles generated by certain intravacuolar bacteria. Although these membrane remnants are known to help microorganisms evade the host’s phagolysosomal pathway, their interaction with Gal-3 enables the recruitment of autophagy adaptor proteins, thereby directing damaged vesicles toward autophagy [27]. Gal-3 is highly expressed in phagocytic macrophages, with its levels increasing as monocytes differentiate into macrophages and decreasing during the maturation of immature dendritic cells [25], which may indicate this glycoprotein as a marker of monocyte-macrophage differentiation and macrophage activation. In addition, widely distributed in lymphoid organs and immune cells, Gal-3 is upregulated in activated CD4^+^ and CD8^+^ T lymphocytes, and its inhibition suppresses T cell proliferation, highlighting its role in promoting inflammation [27]. The effects of Gal-3 on monocyte-monocyte interactions have also been implicated in chronic inflammatory and fibrotic diseases through the formation of multinucleated giant cells [25]. Chronic inflammation can be induced by a variety of stimuli, including persistent infections, autoimmune reactions, allergic responses, chemical insults, radiation, and tissue injury. Persistent inflammation, which is involved in the pathogenesis of many chronic human diseases, can ultimately lead to fibrosis, loss of tissue structure, scarring, and consequent organ failure [28,29,30]. Fibrosis is a major cause of morbidity and mortality worldwide, with limited treatment options; in advanced stages, organ transplantation remains the only effective therapy [25]. Tissue fibrosis is driven by fibroblasts and myofibroblasts, which play central roles in initiating and sustaining fibrogenesis, along with macrophages, which contribute to both the progression and resolution of the process [31,32]. In tissue fibrosis, Gal-3 facilitates the release of pro-fibrotic factors, activates macrophages, can stimulate DNA synthesis in vitro, promotes the proliferation of ECM-producing fibroblasts and myofibroblasts from various tissues, and contributes to tissue damage. It is thought that Gal-3 in the process of fibrosis cross-links with glycans on the TGF-β receptor, leading to sustained receptor activation [25,33]. By linking inflammation-associated macrophages to fibrosis, Gal-3 may be designated as a marker of inflammatory and fibrotic processes rather than a marker of a specific organ, as its circulating sources are often unclear [34].

Evidence from in vitro and in vivo studies shows that inhibition of Gal-3, either by carbohydrate ligands or by gene knockout, significantly reduces fibrosis [33]. In this regard, Gal-3, as a key regulator of inflammation and tissue fibrogenesis, represents an attractive new therapeutic target in the search for effective antifibrotic treatment [35]. The aim of this paper is to comprehensively analyze the molecular and cellular roles of galectin-3 in the pathogenesis of gastrointestinal inflammatory diseases, with a focus on its contribution to immune regulation, cytokine signaling, and fibrotic remodeling, and to evaluate its potential as both a biomarker and therapeutic target.

## 3. Galectin-3 in Inflammatory Diseases of the Gastrointestinal Tract

**Figure 3:**
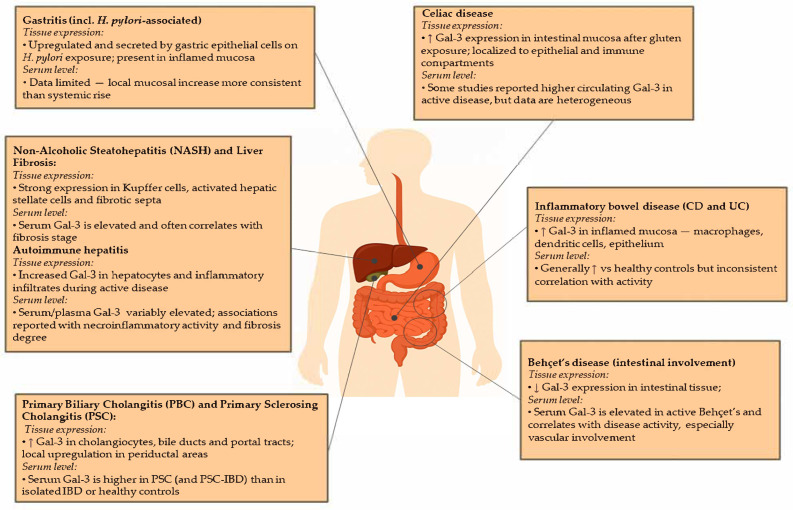
Comparative profiles of Galectin-3 expression in tissue biopsies and serum levels across inflammatory gastrointestinal diseases. ↑—increased; ↓—decreased.

In the gastrointestinal tract, Gal-3 is constitutively expressed by epithelial cells, macrophages, dendritic cells, and fibroblasts, and is upregulated in response to tissue injury and inflammation. Its immunomodulatory properties have been implicated in both protective and pathogenic processes across several inflammatory GI disorders, including gastritis, non-alcoholic steatohepatitis (NASH), autoimmune hepatitis, liver fibrosis, primary sclerosing cholangitis, ulcerative colitis (UC), Crohn’s disease (CD), celiac disease (CeD), and Behçet’s disease (Table 1 and Figure 3).

### 3.1. Galectin-3 in Gastritis

Gastritis is the inflammation of the stomach lining, most often caused by bacteria or frequent use of anti-inflammatory painkillers. Chronic gastritis remains one of the most widespread infections worldwide, with serious consequences such as peptic ulcers and gastric cancer. It has been demonstrated that there is an association between Gal-3 and gastritis caused by *Helicobacter pylori* (*H. pylori*) infection. In vitro, Gal-3 expression has been shown to be increased by gastric epithelial cells following *H. pylori* adhesion, suggesting that in addition to colonization, this protein also plays a role in the host’s response to infection [36]. Also experimental mouse model confirmed a significant role of Gal-3 in innate immunity against infection and colonization of gastric mucosa by *H. pylori*. Specifically, Gal-3 is abundantly secreted by gastric surface epithelial cells into the mucus, where it traps and aggregates *H. pylori*, preventing its deep migration and adhesion to epithelial cells—critical steps for colonization. In Gal-3-deficient mice, *H. pylori* penetrates deeper into gastric glands, and macrophages lacking Gal-3 are less effective at killing the bacteria. Recombinant Gal-3 directly induces aggregation and exhibits bactericidal activity against *H. pylori*, possibly by disrupting its metabolism, as evidenced by increased ATP levels. Notably, Gal-3 did not affect *E. coli*, indicating specificity in its antibacterial action [37]. On the other hand, it has been shown in vitro that administration of extracellular recombinant Gal-3 (rGal-3) inhibits the adhesion of *H. pylori* to gastric epithelial cells leading to a reduction in apoptosis. This biomarker acts as a chemoattractant in the recruitment of THP-1 monocytes, acting as a negative regulator of *H. pylori* infection and its effects on the gastric mucosa [38].

To date, a strong association between *H. pylori* infection and the development of gastric adenocarcinoma and gastric mucosa-associated (MALT) lymphoma has been demonstrated [39,40]. In the light of current knowledge, the potential role of Gal-3 in the process of gastric carcinogenesis is still debatable. In in vitro conditions, it has been shown that Gal-3 expression and upregulation may be a critical endogenous event in *H. pylori* infection that interferes with various intracellular events, causing prolonged cell survival, suggesting carcinogenesis [41]. On the other hand, in vivo studies have shown the formation of submucosal lymphoid clusters predominantly composed of B cells, which may be associated with the pathogenesis of MALT lymphoma, mainly in Gal-3-deficient mice. [37]. Moreover, in WT mice infected with *H. pylori* Sydney strain 1, no MALT lymphoma formation has been observed even after a 2-year follow-up period [42].

### 3.2. Galectin-3 in Non-Alcoholic Steatohepatitis

Non-alcoholic steatohepatitis (NASH) is a condition in which fat accumulates in the liver along with consequent inflammation, composed predominantly of lymphocytes and Kupffer cells, and liver damage, in the absence of a history of alcoholism. It is more common in patients with obesity, metabolic syndrome and type 2 diabetes mellitus and is the most common chronic liver disease, with a rapidly increasing prevalence worldwide. It is characterized by the presence of steatosis on histopathologic material with lobular inflammation, hepatocellular ballooning, and fibrosis, usually beginning in zone 3 and showing a characteristic perisinusoidal/pericellular distribution [43]. There are conflicting data on the association between Gal-3 and NASH. Gal-3 knockout mice (Lgals3^–/–^) have been shown to have less pronounced fat accumulation, oxidative stress, hepatocyte damage, inflammation, fibrosis, and possibly insulin resistance when fed a high-fat diet [44]. These results are also supported by the fact that pharmacologic inhibition of Gal-3 attenuated hepatic steatosis, fat accumulation and insulin resistance in a high-fat diet-induced mouse model of non-alcoholic fatty liver disease (NAFLD) by reducing CD36 expression through suppression of PPARγ [45]. Also, a recent study utilizing a multilineage 3D spheroid model demonstrated that Gal-3 inhibition reduces neutral lipid accumulation and type I collagen levels in vitro [46]. However, another group observed that Gal-3 null mice show an increased tendency to develop NASH [47,48], but there is reduced inflammation, liver cell injury and fibrosis [48]. This view can be explained by the described profibrogenic IL-33/ST2/IL-13 pathway that is dependent on Gal-3 [48]. Namely, IL-33 in vivo administration promoted fibrosis in high-fat diet-induced NASH in all mice, but significantly less in Gal-3 knockout mice, which had fewer IL-13–producing ST2-positive myeloid cells. This suggests that Gal-3 regulates the profibrotic IL-33/ST2/IL-13 pathway in diet-induced NASH [49].

Transcriptomic study in pigs identified LGALS3 as a gene associated with NAFLD progression, suggesting its potential as a biomarker [50]. In children with NAFLD, Gal-3 expression correlated with steatosis and NASH, although macrophage-derived Gal-3 showed an inverse relationship. Mechanistically, Gal-3 promotes NASH progression by activating the NLRP3 inflammasome through TLR4 interaction, linking metabolism and inflammation. However, serum Gal-3 levels have not consistently correlated with disease presence, suggesting its utility may be greater in advanced stages like fibrosis and cirrhosis [51].

Since Gal-3 is perceived as a critical regulator of liver fibrosis, attempts have been made to inhibit its activity using a specific inhibitor, named GR-MB-02, in humans. Doses of GR-MD-02 were shown to be in the upper range of the target therapeutic dose determined from preclinical data and were safe and well tolerated with evidence of a pharmacodynamic effect. These results support a phase 2 development program in advanced fibrosis due to NASH [52].

### 3.3. Galectin-3 and Autoimmune Hepatitis

Autoimmune hepatitis (AiH) is defined as a chronic disease characterized by apoptotic hepatocytes and portal inflammatory reaction inducing the collapse of liver lobules [53]. AiH pathogenesis is initiated by a TH1/TH17 phenotype marked by CD8^+^ T cell infiltration and then switches to a TH2 signature that promotes clonal expansion of autoantibody-secreting plasma cells and inhibits TH1/TH17 functions [54,55,56]. In the course of suppressing tissue inflammation, regulatory T (Treg) cells dampen the immune response and natural killer T (NKT) cells initiate a fibrotic response [57,58]. Gal-3 is capable of inducing hepatocyte apoptosis and liver fibrosis, and orchestrates the activation and differentiation of hepatic stellate cells (HSCs) into myofibroblasts [59,60,61]. Induction of hepatitis by concavalin-A (Con A), which mimics the pathological changes seen in AiH patients, in Gal-3^−/−^ mice resulted in mild liver injury, marked by low levels of proinflammatory cytokines secreted by hypoactive lymphocytes and dendritic cells and high numbers of annexin V + propidium-idodid+ later apoptotic cells [62,63]. More specifically, Gal-3 plays a key proinflammatory role in Con A-induced hepatitis by enhancing T lymphocyte and natural killer T cell activation, promoting dendritic cell maturation, stimulating proinflammatory cytokine production, inhibiting M2 macrophage polarization, and inducing apoptosis of mononuclear cells in the liver [62]. This glycoprotein also induces myofibroblast activation and favors phagocytosis by HSCs during liver fibrosis [60,64], further suggesting that it plays a significant role in the pathogenesis of Con A-induced hepatitis and possibly in AiH [21]. In AiH-associated cirrhosis, Gal-3 levels ≥ 20.95 ng/L were found to be independent risk factor for death, making it an important biomarker for assessing prognosis [65].

### 3.4. Galectin-3 in Liver Fibrosis

Liver fibrosis results from chronic liver injury caused by factors like alcohol consumption, NASH, viral hepatitis (HBV, HCV), autoimmune hepatitis, NAFLD, and cholestatic liver diseases. These conditions trigger chronic inflammation and an abnormal wound-healing response, involving various cells and mediators. Fibrosis leads to excessive ECM accumulation, disrupting liver architecture, causing hepatocyte loss, and impairing liver function, potentially leading to failure. While fibrosis is reversible, progression to cirrhosis makes recovery unlikely. Removing the causative agent can aid fibrosis regression unless advanced cirrhosis has developed [66].

The role of Gal-3 in liver fibrosis was first identified when its upregulation was linked to hepatic fibrosis, and its genetic disruption blocked myofibroblast activation and procollagen (I) expression [60,67]. Further studies confirmed its upregulation in liver fibrosis, NASH, and cholestatic liver diseases. Gal-3, secreted by monocytes and macrophages, activates fibroblasts into myofibroblasts, a key event in fibrosis. Gal-3 is abundant in liver macrophages, with activated macrophages in injured liver tissue showing strong Gal-3 staining. Macrophage plasticity enables shifts between pro- and anti-inflammatory states, with CD68 marking all macrophages and CD206 identifying M2-type macrophages [67]. In pediatric NAFLD, increased α-SMA/Gal-3+ macrophages correlated with fibrosis severity, reinforcing Gal-3′s pro-fibrotic role. Recent studies using RNA sequencing identified TREM2+ CD9+ NASH-associated macrophages (NAMs) that expand in fibrosis and share similarities with lipid-associated macrophages (LAMs) in adipose tissue. Live cell-to-cell mapping confirmed Gal-3 glycan interactions among hepatocytes, macrophages and HSCs, while genomic studies in a swine NASH model linked LGALS3 to lipid droplet accumulation, highlighting its role in NAFLD progression [67]. More precisely, Gal-3 aids in clearing advanced glycation and lipidomic end products in the liver, and its absence leading to higher circulating levels of these toxic by-products [68].

In a liver fibrosis model, Gal-3 expression closely aligned with myofibroblast activation and collagen deposition. As an immediate early gene, Gal-3 rapidly increases in response to injury. It was found that HSC activation occurred in WT but not Gal-3 KO HSCs, though this was restored by adding exogenous Gal-3. Small interfering RNA (siRNA) knockdown of Gal-3 blocked WT HSC activation, indicating that Gal-3 autocrine signaling is sufficient for HSC activation in vitro. In vivo, HSCs interact with Kupffer cells, endothelial cells, and hepatocytes, facilitating both autocrine and paracrine activation via cell contacts and soluble factors. Additionally, injured hepatocytes and macrophages upregulate Gal-3, further driving HSC activation and fibrosis [60]. Finally, in an integrated transcriptomic, proteomic, and in vivo mouse model analysis, it has been shown that increased secretion of Gal-3-binding protein (LGALS3BP) plays a pivotal role in activating TGF-β1 signaling, a pathway essential for controlling liver inflammation, fibrosis, and cancer development [69].

It has been shown that Gal-3 levels increase in advanced liver fibrosis (F3/F4) compared to early stages (F0/F1) and are elevated in alcoholic liver disease, correlating negatively with liver function. In alcoholic cirrhosis, Gal-3 levels are highest and align with the Child–Pugh score [70].

Gal-3′s role in hepatic immunopathology caused by *Schistosoma japonicum* (*S. japonicum*) infection has also been highlighted. Blocking Gal-receptor interactions improved liver function, reduced pathology, and decreased fibrosis in infected C57BL/6 mice, suggesting a potential therapeutic approach. Compared to untreated infected mice, those with blocked Gal-3 interactions exhibited lower Gal-3 expression, reduced liver fibrosis markers (α-SMA, collagen I/IV), and decreased *S. japonicum* egg burden. Immunofluorescence and flow cytometry confirmed increased M1 macrophage polarization, further linking Gal-3 to liver fibrosis and immune regulation in *S. japonicum* infection [71].

### 3.5. Galectin-3 in Primary Sclerosing and Primary Biliary Cholangitis

Gal-3 exhibits a complex, context-dependent role in immune-mediated cholangiopathies, particularly primary sclerosing cholangitis (PSC) and primary biliary cholangitis (PBC).

Primary sclerosing cholangitis (PSC) is a chronic liver disease marked by ongoing inflammation and fibrosis of the intrahepatic and extrahepatic bile ducts, resulting in cholestasis, progressive liver scarring, and, in many cases, decompensated cirrhosis within 10–15 years [72]. In one study that aimed to clarify whether Gal-3 can differentiate between patients with IBD, PSC, and PSC-IBD, serum and urinary Gal-3 levels were elevated in PSC patients, especially those with concomitant IBD, compared to IBD alone [73]. Therefore, Gal-3 may have a protective role in IBD, and its elevated levels in PSC-IBD patients could help explain the generally milder IBD symptoms observed in this subgroup [74]. Interestingly, serum and urinary Gal-3 levels showed no correlation with elevated aminotransferases but were strongly linked to PSC. Given the small patient cohort in this study [73], further research is needed to clarify the potential of serum Gal-3 as a noninvasive biomarker for distinguishing isolated PSC from PSC-IBD.

Primary biliary cholangitis is a chronic autoimmune liver disease marked by lymphocytic inflammation and destruction of small bile ducts, typically associated with the presence of antimitochondrial antibodies [75]. In mouse models of xenobiotic-induced autoimmune cholangitis, Gal-3 deficiency significantly increased periportal inflammation, bile duct damage, granuloma formation, fibrosis, and heightened apoptosis in biliary epithelial cells (BECs), alongside elevated infiltration of pro-inflammatory lymphocytes and dendritic cells—indicating a protective function of Gal-3 in preventing biliary epithelial destruction [76].

In a separate *Novosphingobium aromaticivorans*-induced autoimmune cholangitis model, Gal-3 promoted dendritic cell and macrophage activation, triggering IL-17-producing NK, NKT, and T cells—a pathway critical for PBC pathogenesis. Notably, Gal-3 deficiency in this setting suppressed inflammasome activation and IL-17 responses, suggesting potential for therapeutic intervention through Gal-3 inhibition [77].

It has recently been shown that in immune-driven cholestatic liver pathology, Gal-3 interacts with NLRP3 to activate macrophage inflammasomes, leading to pro-inflammatory cytokine release that injures BECs. Given that NLRP3 is also expressed in BECs and that its downregulation may be associated with the development of cholangiocarcinoma, the elevated Gal-3 observed in BECs during PBC suggests a potentially broader role in biliary cell inflammation and tumorigenesis [78].

Gal-3 has also been identified as an autoantigen in IgG4-related disease, with high serum and tissue levels that persist during glucocorticoid therapy. While anti–Gal-3 autoantibodies, mainly of IgG4 and IgE isotypes, are present in IgG4-related disease, they are not observed in PSC, suggesting differential immunopathological roles. In vitro studies with antibodies against Gal-3 (and prohibitin-1) failed to demonstrate increased bile acid permeability in cholangiocytes, implying that these autoantibodies may contribute more to immune dysregulation or cholestatic injury rather than directly damaging cholangiocytes [79].

Collectively, these findings underscore Gal-3′s dual function: it may serve as both a pro-inflammatory mediator and a protective factor in biliary epithelial homeostasis, depending on the disease context and model. Continued research is warranted to clarify its cell-specific and phase-dependent functions and to explore Gal-3 inhibition as a therapeutic strategy in cholestatic liver diseases.

### 3.6. Galectin-3 and Celiac Disease

Celiac disease (CeD) is an autoimmune condition that occurs in genetically predisposed individuals upon gluten intake, primarily targeting the small intestine and resulting in nutrient malabsorption. The pathological process is primarily initiated by gliadin, a protein component of gluten that acts as a key trigger [80]. Although most research in CeD has focused on Gal-1, which increases following adherence to a gluten-free diet in treated CeD patients, similar homeostatic roles are plausible for Gal-3 in mucosal immune regulation. In murine model, Lgals3^−^/^−^ (Gal-3 knockout) mice exposed to oral gliadin developed markedly more severe inflammation throughout the small and large intestine compared to wild-type controls. Findings included increased crypt hyperplasia, intraepithelial lymphocyte infiltration, villus disorganization, and accumulation of apoptotic bodies in Peyer’s patches—features reminiscent of CeD pathology. These results indicate that Gal-3 helps maintain epithelial integrity, mucosal structure, and immunological homeostasis upon gluten exposure [81].

### 3.7. Galectin-3 and Inflammatory Bowel Diseases

Inflammatory bowel diseases, including Crohn’s disease (CD) and ulcerative colitis (UC), are chronic gastrointestinal disorders with unknown etiology, influenced by immune, environmental, and genetic factors. While the role of galectins in IBD remains unclear, these glycan-binding proteins are widely expressed in intestinal cells and have been implicated in intestinal inflammation and immune modulation [82,83]. Previous studies reported changes in Gal-1, -3, -4, and -9 expression in inflamed tissues of IBD patients, distinguishing IBD from other intestinal disorders like CeD. In early research, elevated serum Gal-3 in IBD patients regardless of disease activity has also been found. Recently, significantly increased serum levels of Gal-1 and -3 in IBD patients have been shown, while Gal-2, -4, -7, and -8 remained unchanged. However, these elevated levels did not differentiate active from inactive UC and CD. Even more, combining Gal-1 and -3 with CRP also failed to improve disease activity discrimination. Therefore, given the fact that Gal-1 and -3 levels are elevated in the circulation of IBD patients and may contribute to disease progression, they could serve as general biomarkers of IBD rather than indicators of disease activity [84]. On the other hand, it has been shown that expression profiles of Gal-1, Gal-3, Gal-4, and Gal-9 at mRNA level can distinguish active IBD from non-inflamed or quiescent mucosa. Although no single galectin has been a definitive biomarker, integrated analysis predicted inflammation in 82% of active IBD cases. These proteins have been homogeneously expressed in healthy colon tissue but deregulated in inflamed areas, which highlight galectins as potential mucosal markers for IBD severity assessment [82].

It has been shown that although Gal-3 expression is not significantly different between healthy individuals and IBD patients in remission, it is notably reduced in actively inflamed mucosa. This downregulation, observed in both CD and UC, appears to be a consequence of inflammation rather than a disease-specific trait. In vitro exposure of healthy biopsies to TNF similarly reduced Gal-3 mRNA, suggesting TNF-driven regulation. Given Gal-3′s role in cell adhesion, its reduction may impair the intestinal barrier, contributing to disease pathology. However, the absence of spontaneous inflammation in Gal-3 knockout mice indicates that Gal-3 loss alone does not trigger disease. Gal-3 has dual immunoregulatory functions—protecting T cells from apoptosis intracellularly while promoting apoptosis extracellularly. In IBD, its reduced expression may support T cell activation and prolong immune responses, potentially leading to chronic inflammation if triggered by commensal bacteria or food antigens. Overall, Gal-3 downregulation in inflamed mucosa may initially aid immune activation but could contribute to disease persistence in IBD [85]. The diverse roles of Gal-3 in both innate and adaptive immunity suggest that it functions as an immunoregulatory, rather than purely proinflammatory, factor in the development and progression of IBD [74].

#### 3.7.1. Galectin 3 in Crohn’s Disease

Crohn’s disease, an autoimmune-mediated form of IBD, arises from a combination of genetic susceptibility and environmental triggers that culminate in an abnormal T-cell-driven immune response [86]. Within the intestinal mucosa, Gal-3 released by resident macrophages and colonic epithelial cells stimulates atypical fibroblasts in the lamina propria, promoting NF-κB-dependent IL-8 secretion and initiating fistula development [87]. As the disease progresses, Gal-3 expression in the colon decreases, impairing fibroblast migration into the fistulas [88]. Furthermore, diminished epithelial Gal-3 expression has been strongly linked to epithelial barrier disruption and increased TNF levels [89], suggesting that Gal-3 plays an important role in maintaining epithelial integrity and regulating cell–cell adhesion [21]. Interestingly, elevated serum levels of IgG anti-Gal-3 autoantibodies in CD patients have been negatively correlated with clinical disease activity [90]. On the other hand, high levels of serum Gal-3 and high numbers of CD14+ cells in the blood of patients with CD have also been found during the active stages of the disease [83], suggesting that Gal-3 may have a pro-inflammatory effect in CD and may play a role in the development of the disease [21].

#### 3.7.2. Galectin 3 in Ulcerative Colitis

Ulcerative colitis is a form of IBD marked by continuous, diffuse inflammation confined to the mucosa of the colon, extending proximally from the rectum [91]. Gal-3 plays a dual role in UC, influencing both macrophage activation and T cell responses. In colonic macrophages, Gal-3 promotes inflammation by activating the NLRP3 inflammasome, while its absence reduces cytokine production and alleviates colitis [92]. In addition, animal studies suggest that soluble Gal-3 from colon epithelial cells also activates fibroblasts, driving NF-κB activation and IL-8 secretion [84]. In dextran sulfate sodium (DSS)-induced colitis models, macrophages, a major source of Gal-3, exhibited increased expression, underscoring its role in inflammation [83]. On the other hand, another in vivo study showed that Gal-3 knockout mice exhibited more severe disease activity in the DSS-induced colitis model, suggesting a protective role of Gal-3 against inflammation in this type of colitis [93]. In addition, it has been reported that recombinant Gal-3 induces an immunosuppressive phenotype in T cells, inhibiting their proliferation. Also, adoptive transfer of Gal-3-treated T cells has been shown to significantly reduce chronic colitis in mice [94]. Recently, the exact mechanism by which Gal-3 regulates the immunosuppressive function of regulatory dendritic cells in the gut has been described. Specifically, this biomarker regulates the immunosuppressive function of regulatory dendritic cells in the gut primarily through TLR-4-dependent activation of the IDO-1/KYN pathway, leading to the expansion of colon-infiltrating T regulatory cells that suppress Th1- and Th17-mediated colon inflammation [94]. Finally, while some reports suggest reduced galectin expression in intestinal inflammation, it has also been shown that galectin expression in colonic epithelium was not significantly different between the control group and UC patients with normal histology [95].

#### 3.7.3. Galectin-3 as Biomarker in IBD

Galectin-3 (Gal-3) has attracted attention as a blood and tissue biomarker in IBD: tissue mRNA profiling can help distinguish IBD from other intestinal inflammatory disorders (e.g., CeD), while circulating Gal-3 (together with Gal-1) is reproducibly higher in IBD patients than in healthy controls, although it does not reliably separate CD from UC or active from quiescent disease [84]. Serum Gal-3 at a cutoff of 38.5 ng/mL discriminated IBD from healthy controls with moderate sensitivity and good specificity (53% and 87%, respectively), whereas in one UC cohort ROC analysis indicated that Gal-3 may stratify severity with very high performance (sensitivity 95%, specificity 91.7%), and reported fecal Gal-3 cutoffs were also proposed for distinguishing disease states [84,94]. Practical comparisons with established biomarkers show that CRP and fecal calprotectin remain more widely validated for monitoring systemic and intestinal inflammation, respectively, but both have limitations (CRP lacks specificity; fecal tests have variable patient acceptance and specificity), so Gal-3 and related lectins currently appear most useful as complementary, non-invasive markers that may add diagnostic or staging information when combined with other measures or when standardized cutoffs are validated in large cohorts [96,97]. In summary, reported data support Gal-3 as a promising adjunct biomarker and potential therapeutic target in IBD, but assay standardization, larger prospective validation, and clarification of context-dependent (pro- vs anti-inflammatory) roles are needed before routine clinical implementation [74,84,94,96,98].

### 3.8. Galectin-3 in Behçet’s Disease

Behçet’s disease (BD) is a rare yet highly debilitating vasculitis, primarily presenting with mucocutaneous symptoms such as orogenital ulcers and skin lesions [99]. Significantly lower levels of Gal-3 have been observed in intestinal BD colon tissues, a notable finding given the role of Gal-3 as a regulator of immune response in other chronic inflammatory diseases, including IBD. Using patient tissues, HT-29 cells, and murine bone marrow derived macrophages, it has been demonstrated that Gal-3 can modulate ER stress, autophagy, and inflammasome activation, suggesting a potential protective role of Gal-3 in intestinal BD. More specifically, it was shown that the expression of TGF-β and IL-10 was significantly lower in stable Gal-3-silenced (shLGALS3)-transfected cells, while expression of GRP78 and XBP1s and apoptosis rates were all higher following the induction of ER stress [100].

### 3.9. Context-Dependent Effects of Galectin-3 in Gastrointestinal Inflammation

The seemingly contradictory reports of Gal-3 as a harmful or protective agent in some inflammatory gastrointestinal diseases, such as NASH, CD, or UC, are not irreconcilable; rather, they reveal context-dependent pleiotropy. Gal-3 is a multifunctional lectin whose specific effect in the aforementioned diseases depends on (i) the dominant producing cell type and whether Gal-3 acts intra- or extracellularly, (ii) the disease phase (induction vs. resolution),(iii) experimental approach (genetic knockout vs. acuteinhibition vs. recombinant addition), (iv) glycan ligand context and microbiome composition, and (v) the specific outcome measured (inflammation, barrier function, fibrosis). Reconciling conflicting data therefore requires experimental designs that (a) distinguish intracellular from extracellular Gal-3 actions, (b) manipulate Gal-3 in a cell-type-specific and temporally controlled manner, and (c) report multiple outcome domains (metabolic effects, inflammation, barrier integrity, repair/fibrosis) instead of a single endpoint. Studies that meet these criteria will most likely define when Gal-3 is a valid therapeutic target versus when modulation would be detrimental [43,44,45,46,47,48,49,82,83,84,92,93,94].

## 4. Galectin-3 as a Targeted Therapy in Inflammatory Diseases of Gastrointestinal Tract

Gal-3 is a long-standing drug target, with most pharmacological strategies focused on its CRD in complex with lactose, as this protein–sugar interface drives its biological functions. Natural ligand binding is mediated by hydrogen bonds and van der Waals forces, but these interactions are relatively weak, leading to low binding affinities. Structure-based drug design has enhanced potency and selectivity by adding aromatic substituents to galactose cores, enabling stronger interactions, especially π-stacking with Arg144, a residue absent in most other galectins. Some potent inhibitors also target Arg186, though this residue is more conserved, requiring careful design to balance potency and selectivity. Advances in crystallography, including neutron studies, have clarified binding thermodynamics and guided the development of selective inhibitors like GB0139 (inhaled) and GB1211 (orally bioavailable), along with novel chemoenzymatic lactose derivatives. Recently, non-sugar-based inhibitors, such as MG-257, have emerged, showing promise for strong and stable binding to Gal-3, though structural confirmation of these interactions is ongoing [101].

Gal-3 inhibitors are emerging antifibrotic agents targeting chronic inflammation and fibrogenesis (Table 2) [102]. Development efforts have explored monoclonal antibodies, natural galactose-based polymers (e.g., pectins), synthetic multivalent ligands, and small molecules. However, many currently available agents suffer from poor cellular uptake, low binding affinity, or limited oral bioavailability, and evidence for pectins’ Gal-3 inhibition remains weak [103].

Belapectin (galactoarabino-rhamnogalacturonate [GR-MD-02]) is a plant-derived complex carbohydrate containing galactose-rich oligosaccharide chains that bind primarily to Gal-3 and, to a lesser extent, Gal-1 [104]. The first human trial (2016) tested GR-MD-02 (belapectin) in NASH patients with bridging fibrosis (F3) to assess safety, tolerability, and pharmacokinetics. In this placebo-controlled, double-blind study, patients received 2, 4, or 8 mg/kg doses. All doses were safe and well-tolerated, with no significant adverse events. While small sample sizes in lower-dose groups limited statistical analysis, the 8 mg/kg group showed a significant reduction in FibroTest scores [52]. A subsequent phase IIb trial in NASH cirrhosis with portal hypertension (hepatic venous pressure gradient (HVPG) ≥ 6 mmHg) compared biweekly infusions of 2 mg/kg, 8 mg/kg, or placebo over 52 weeks. The primary endpoint (HVPG reduction) and most secondary endpoints (fibrosis, NAFLD score, liver outcomes) were not met. However, a subgroup without esophageal varices receiving 2 mg/kg showed reduced HVPG and prevention of new varices. Overall, GR-MD-02 was safe and well-tolerated, but efficacy was limited to specific patient subgroups [104]. The ongoing phase 3 NAVIGATE trial is evaluating its safety and efficacy, with primary endpoints including variceal development and event-free survival (NCT04365868).

Selvigaltin (GB1211) is a novel small-molecule Gal-3 inhibitor from a new class of α-D-galactopyranosides with aromatic substitutions at the 1- and 3-positions, optimized through specific interactions such as fluorine-amide and halogen bonding [103]. GB1211 is a highly selective, orally bioavailable Gal-3 inhibitor, primarily synthesized for the potential treatment of cancer and fibrotic disorders, including liver cirrhosis [103,105,106]. In vitro, it reduced Gal-3 expression in human macrophages and suppressed TGF-β-induced pro-fibrotic gene expression in human stellate cells. In mouse models of CCl_4_-induced liver fibrosis and bleomycin-induced lung fibrosis, GB1211 demonstrated antifibrotic effects with therapeutic dosing. Following a phase I trial evaluating safety, tolerability, pharmacokinetics, and biomarkers in healthy volunteers (NCT03809052), GB1211 was selected for phase IIa development as a potential treatment for hepatic impairment (NCT05009680) [51]. In this study, it was shown that hepatic impairment increases selvigaltin exposure, but no adverse events were reported [107].

Olitigaltin (GB0139, TD139) [108] is a high-affinity 3,3′-bis-(4-aryltriazol-1-yl) thio-digalactoside inhibitor of the Gal-3 carbohydrate recognition domain, effective in murine lung fibrosis models. Its antifibrotic action stems from blocking Gal-3-secreting macrophage recruitment and expansion, thereby limiting myofibroblast activation. Preclinically, GB0139 modulates macrophage phenotype and Gal-3 expression, suppresses fibroblast activation and profibrotic growth factor effects, and inhibits epithelial–mesenchymal transition [109]. In liver disease models, GB0139-mediated Gal-3 inhibition reduced fatty acid accumulation in early-stage NAFLD in mice. In a glucocorticoid-induced liver injury model, GB0139 preserved hepatic progenitor cells and improved liver function. However, its high polarity limits oral bioavailability, restricting its potential for systemic use [51].

GB1107 is orally active 1,3-substituted-α-D-monogalactopyranosides with high Gal-3 affinity [110]. Because GB1107 can penetrate cells, it may also target cytosolic Gal-3, unlike most inhibitors that act only on the extracellular form. X-ray crystallography has shown that GB1107 binds specifically to the carbohydrate-binding site within Gal-3’s CRD [111]. In early research, GB1107 showed, compared to GB1211, higher Gal-3 affinity and pharmacokinetic performance in mice, enabling once-daily dosing and effective vehicle formulation, but was not advanced clinically due to hERG toxicity [105]. Recently, it has been shown in a mouse model that GB1107 significantly lowers plasma transaminases, hepatic Gal-3 levels, and liver fibrosis. RNA sequencing of whole liver tissue revealed 1659 differentially expressed genes (DEGs) in the CCl_4_-treated group versus controls, with upregulated pathways linked to extracellular matrix remodeling, collagen biosynthesis and assembly, cell cycle regulation, and immune responses. In GB1107-treated mice, 1147 DEGs were identified compared to the CCl_4_ group, with GB1107 reversing most of the gene expression changes induced by CCl_4_ [110].

The following table summarizes current therapeutic evidence for Gal-3 inhibition in experimental and clinical models of gastrointestinal and liver inflammatory diseases, underlining its promise as a novel anti-inflammatory and anti-fibrotic strategy.

**Table 1:** Overview of Gal-3 roles, molecular mechanisms, and effects in inflammatory diseases of the gastrointestinal tract.

Disease	Role of Gal-3	Mechanism/Pathway	Effect	Refs.
Gastritis/*H. pylori*	Induced by *H. pylori* infection—a role in the host’s response to infection	Enhances innate immune response and monocyte recruitment and reduces bacterial adhesion and epithelial cell apoptosis	Initially contributes to host defense, while under chronic conditions, could potentially promote gastric carcinogenesis	[36,37,38,39,40,41]
Non-alcoholic Steatohepatitis ^1^	Regulator of fat metabolism in the liver; mediator of hepatic inflammation and fibrosis	Induces HSC activation, regulates IL-33/ST2/IL-13 profibrotic pathway and PPARγ/CD36 signaling pathway, increases oxidative stress	Drives progression from steatosis to NASH (promotes lipid accumulation in the liver), liver fibrosis and hepatocellular carcinoma	[43,44,45,46,47,48,49]
Autoimmune Hepatitis	Involved in the pathogenesis of AiH—proinflammatory role	Promotes activation of T and NK T cells, enhances dendritic cell maturation, increases proinflammatory cytokines, suppresses M2 macrophage polarization, and induces mononuclear cell apoptosis	Promotes hepatic injury and autoimmune inflammation	[57,58,59,60,61,62,63,64]
Liver Fibrosis	Fibrogenic activator via macrophages and HSCs	Activates TGF-β1 pathway; modulates ECM remodeling	Induces and maintains fibrotic response	[67,68,69,70]
Primary Sclerosing Cholangitis	Proinflammatory role	Not described	Contributes to bile duct fibrosis and inflammation	[72,73,74]
Primary biliary cholangitis	Involved in biliary inflammation	Binds to NLRP3 and activates macrophage inflammasomes	Release of pro-inflammatory cytokines that affect integrity of BECs and triggers their damage	[75,76,77,78]
Celiac Disease	Protective role during intestinal inflammation	Ameliorates crypt hyperplasia, intraepithelial lymphocyte infiltration, villus disorganization, and accumulation of apoptotic bodies in Peyer’s patches	Regulates gut homeostasis under physiologic conditions - maintains epithelial integrity, mucosal structure, and immunological homeostasis	[81]
Crohn’s Disease ^1^	Immunoregulatory role	NF-κB/IL-8 pathway activation	Suppresses/Enhances chronic intestinal inflammation	[83,86,87,88,89,90]
Ulcerative Colitis ^1^	Immunoregulatory role	NLRP3 inflammasome activation; NF-κB activation and IL-8 secretion,; TLR-4-dependent activation of IDO-1/KYN pathway	Reduces/increases inflammation	[84,92,93,94]
Behçet’s Disease (GI Involvement)	Protective role	Regulates TGF- β and IL-10 release and expression of GRP78 and XBP1s	Modulation of ER stress, autophagy, and inflammasome activation	[100]

^1^ The results are still contradictory and require further research.

**Table 2:** Current status of key Gal-3 inhibitors in the treatment of liver fibrosis.

Inhibitor	Type	Stage	Key Findings	Refs.
Belapectin(GR-MD-02)	Plant-derived polysaccharide	Phase III (NAVIGATE)	Safe; limited efficacy, benefit in subgroup without varices	[52,104]
Selvigaltin(GB1211)	Small molecule, orally bioavailable	Phase IIa	Reduced Gal-3 activity; well tolerated; clinical efficacy pending	[51,103,105,106,107]
Oltigaltin(GB0139/TD139)	High-affinity thiodigalactoside(inhaled)	Preclinical	Antifibrotic in models; poor oral bioavailability	[51,108,109]
GB1107	Orally active monogalactopyranoside	Preclinical	Strong antifibrotic effects; discontinued due to toxicity	[105,110,111]

## 5. Conclusions and Future Perspectives

This review is an attempt to describe inflammatory diseases in gastroenterology that present diagnostic as well as therapeutic challenges, in which the role of Gal-3 has been assumed or demonstrated. Knowledge of the role of Gal-3, its mechanism of action, as well as the mechanism of its inhibition, gives gastroenterologists hope that the discovery of a revolutionary biomarker is on the horizon, which can not only serve in the prognosis, but also in the treatment of numerous gastroenterological diseases, which by their nature are usually chronic, progressive and resistant to therapeutic modalities. The discovery of such a biomarker would not only enable the diagnosis of the disease in its earlier stage, but also modulate the course of the disease or slow down its progression.

In the future, research focus should be on developing selective Gal-3 inhibitors and evaluating them in preclinical and clinical trials across different gastrointestinal disorders. Longitudinal studies are needed to determine whether serum Gal-3 levels can predict disease progression, relapse, or response to treatment. Integration of Gal-3 profiling with other biomarkers and imaging techniques may help establish personalized approaches to patient care. Advances in molecular biology and high-throughput screening could uncover novel regulatory pathways involving Gal-3, offering additional therapeutic targets. Finally, collaborative efforts between fundamental scientists, clinicians, and pharmaceutical companies will be essential to translate Gal-3 research into effective clinical applications.

## Data Availability

No new data were created or analyzed in this study.
